# *In situ* Protein Detection for Companion Diagnostics

**DOI:** 10.3389/fonc.2013.00271

**Published:** 2013-10-31

**Authors:** Gabriela Gremel, Karin Grannas, Lesley Ann Sutton, Fredrik Pontén, Agata Zieba

**Affiliations:** ^1^Science for Life Laboratory, Department of Immunology, Genetics and Pathology, Uppsala University, Uppsala, Sweden

**Keywords:** companion diagnostics, immunohistochemistry, Her2, alternative binders, proximity ligation assays

## Abstract

The emergence of targeted therapies for cancer has created a need for the development of companion diagnostic tests. Assays developed in recent years are aimed at determining both the effectiveness and safety of specific drugs for a defined group of patients, thus, enabling the more efficient design of clinical trials and also supporting physicians when making treatment-related decisions. Immunohistochemistry (IHC) is a widely accepted method for protein expression analyses in human tissues. Immunohistochemical assays, used to localize and quantitate relative protein expression levels within a morphological context, are frequently used as companion diagnostics during clinical trials and also following drug approval. Herein, we describe established immunochemistry-based methods and their application in routine diagnostics. We also explore the possibility of using IHC to detect specific protein mutations in addition to DNA-based tests. Finally, we review alternative protein binders and proximity ligation assays and discuss their potential to facilitate the development of novel, targeted therapies against cancer.

## Introduction

Throughout recent decades, our understanding of the molecular basis of cancer development has dramatically improved. This is reflected in the growing number of targeted cancer therapies and significantly affects today’s standard of care in oncology. Nevertheless, a prerequisite for an effective, targeted cancer treatment concerns the selection of patient, which creates a growing demand for reliable companion diagnostic devices. The rationale behind such developments is to ensure that treatment is not withheld from patients whom it may benefit while at the same time protecting them from overtreatment, the risk of unnecessary side effects and, most importantly, a delay in receiving treatment with a more suitable agent.

Companion diagnostics also play an important role during the pre-clinical stages of drug testing. A potent effect observed in a small patient population may be missed by the absence of a reliable companion diagnostic test. Conversely, a novel subset of patients may be found to benefit from treatment or no difference in efficiency may be detected, regardless of biomarker positivity. These issues pose a challenge to the parallel development of drug and companion diagnostic tests and consequently, the latter should be fully validated before the initiation of clinical trials and the trial design adjusted accordingly (1). Despite much advances, the corresponding regulatory framework is still incomplete and while the US Food and Drug Administration (FDA) dictates a stringent premarket approval procedure for all companion diagnostic devices, similar legislation is still under review in Europe (1). Currently, only 19 companion diagnostic devices have been approved by the FDA, 10 of which are intended for the detection of the human epidermal growth factor receptor 2 (ERBB2, also referred to as HER2)^1^.

Most companion diagnostic tests used in a clinical setting are based on immunohistochemistry (IHC), real-time reverse transcription PCR (qRT-PCR), or *in situ* hybridization (ISH). With regard to ISH, assay systems based on either fluorescent (FISH) or colorimetric (CISH) signal detection have been established and each testing modality is associated with a number of advantages and disadvantages (Table 1).

**Table 1:** **Advantages and disadvantages of currently used companion diagnostic techniques**.

Technique	Advantage	Disadvantage
IHC	Routinely performed; low technological requirements; time and cost effective; preservation of histological information; suitable for small tumor samples	Semi-quantitative; subjective interpretation of results; variability dependent on fixation procedure, staining protocol, and antibody selection
qRT-PCR	Quantitative; large dynamic range	No histological information retained; contamination of test results by stromal/normal tissue possible; increased technological requirements; increased time and cost requirements; variability dependent on tissue quality, RNA extraction/processing procedures and primer/probe selection
ISH	Quantitative for genetic alterations; higher reproducibility	Increased technological requirements (especially for FISH); increased time and cost requirements; added expertise in result interpretation necessary

IHC, immunohistochemistry; qRT-PCR, quantitative real-time PCR; ISH, *in situ* hybridization; FISH, fluorescent *in situ* hybridization; CISH, colorimetric *in situ* hybridization.

While the scope of companion diagnostics is broad, with this review we will focus on techniques designed to detect proteins in formalin-fixed, paraffin-embedded (FFPE) tissue. We will discuss currently applied companion diagnostic tests which use IHC and also novel developments regarding mutation-specific antibodies, *in situ* proximity ligation assays (PLA), and alternative protein binders.

## Application of Immunohistochemistry in Clinically Used Companion Diagnostics

### Estrogen receptor

The introduction of tamoxifen, a selective estrogen receptor (ER) modulator, over 30 years ago has revolutionized the clinical management of breast cancer. However, since significant treatment benefits were only observed in ER-positive patients (2, 3), companion diagnostic testing became imperative. Originally, various ligand binding assays (LBAs) were used to quantify the expression of ER, however, they required homogenization of fresh frozen tumor material and were thus laborious in their execution. With the development of monoclonal antibodies targeting the ER and antigen retrieval methods for the use of FFPE tissue, LBAs were soon replaced and IHC became the standard diagnostic tool. Several grading systems were subsequently introduced to describe IHC-based ER expression levels, including (1) Allred score (range: 0–8) (4), (2) Quick score (range: 0–7) (5) (both of which are based on the sum of fraction and intensity units of the stained cells), (3) *J*-score (range: 0–3, based on the fraction of stained cells) (6) and (4) H-score (range: 0–300, based on the product of fraction and the intensity unit of stained cells) (7). Differences between positive/negative definitions together with variations regarding antibody clones, tissue fixation, antigen retrieval, and detection protocols, all contributed to a significant rate of variability in ER detection (8–10). For instance, a study headed by the UK National External Quality Assessment Scheme for Immunocytochemistry (NEQAS-ICC) reported false negativity rates ranging from 30 to 60% following the testing of a standardized sample with low ER expression by 200 laboratories throughout 26 countries (8).

Due to the historic nature of ER testing, there are currently no FDA-approved companion diagnostic devices available. To minimize inter-laboratory variation, the American Society of Clinical Oncology and the College of American Pathologists (ASCO/CAP) have recently published a document outlining their recommendations for the immunohistochemical testing of ER in breast cancer (11). The optimal testing conditions and tissue handling requirements were defined together with guidelines for both the internal and external quality assurance procedure. The same guidelines were also applicable to the detection of the progesterone receptor (PR) via IHC. PR is located downstream of ER and a positive PR test result may be indicative of an intact estrogen signaling cascade (12). This line of thinking was corroborated by the finding that patients with PR positive tumors had a better prognosis than patients with ER-positive/PR negative breast cancers (13). Alternative methods for the detection of ER and PR are continuously under investigation and Oncotype DX is one such example. This is a qRT-PCR-based assay system designed to estimate the probability of distant tumor recurrence in tamoxifen-treated, node-negative breast cancers (14) and measures the expression PR and ER, together with 19 other genes, in mRNA extracted from FFPE tissue. Proof-of-concept studies on the applicability of Oncotype DX as a companion diagnostic tool for tamoxifen treatment reported varying conclusions. In a study supported by Genomic Health Inc., Badve and colleagues reasoned that ER/PR determination using Oncotype DX performed comparably well to IHC-based detection systems (15). This was in contrast to an independent evaluation by Kraus et al. who concluded that IHC was superior to the Oncotype DX qRT-PCR-based test, not only due to higher sensitivity but also the lower cost, the ease of application and the preservation of morphological information (16).

### Human epidermal growth factor receptor 2

Overexpression of the HER2 protein and/or amplification of the HER2-encoding gene have been associated with an unfavorable prognosis in several types of cancer, including breast, gastric, and pancreatic cancer (17–19). Trastuzumab (Herceptin), an antibody-based inhibitor, was the first HER2-targeted drug to be approved for the treatment of breast cancer and has since been shown to significantly improve survival in a metastatic and adjuvant setting (20, 21). Numerous studies support the close relationship between HER2 positivity and trastuzumab responsiveness (20, 22) and bearing in mind that a HER2-amplification rate of approximately 25% occurs in breast cancer, initial clinical trials may not have yielded significant data had no pre-selection of patients according to HER2 status taken place (23). Since gene-amplification is the primary cause of HER2 overexpression (24), both FISH- (or CISH-) and IHC-based companion diagnostic devices have been approved by the FDA. Nevertheless, the initial evaluation of HER2 status is usually performed using an IHC-based method and only ambiguous (or equivocal) cases are subjected to FISH reflex testing.

Substantial inter-laboratory variations in test results are an inherent problem when considering IHC-based tests. Similar to the guidelines established for ER, ASCO/CAP has produced recommendations for HER2 testing in breast cancer (25, 26). In contrast to the instructions for the commonly used FDA-approved HercepTest (Dako), which states that a finding of more than 10% of cells with strong, uniform membrane staining qualifies as a positive result, the ASCO/CAP guidelines require complete intense membrane staining in >30% of cells in order to qualify as a positive test result following IHC testing. In addition, unlike the FDA-approved cut-off ratio of 2.0 for HER2/Chromosome 17 centromere (CEP17) testing via FISH (or four HER2 copies in assays without internal CEP17 probes), ASCO/CAP considered the range of ratios between 1.8 and 2.2 (or four to six HER2 copies) as equivocal and stated that only cases with HER2/CEP17 ratios >2.2 (or more than six copies of HER2) could be deemed to be positive based on FISH analysis. The aim of the new ASCO/CAP guidelines was to reduce the number of inconclusive cases and although some groups were positive to these new definitions (27, 28), others saw no added benefit (29). While high concordance between IHC- and FISH-based HER2 testing was demonstrated by several studies, thus justifying the use of routine IHC as an initial test (30), critics are keen to highlight the technical superiority of FISH over IHC and consequently advocate FISH as the gold standard for HER2 testing (31). Hence, it is evident that more conclusive studies on the clinical significance of both testing modality are required.

### Epidermal growth factor receptor

The epidermal growth factor receptor (EGFR) is a prominent therapeutic target in both colorectal and non-small cell lung cancer (NSCLC). In colorectal cancer, it is primarily targeted by the monoclonal antibody-based drugs cetuximab and panitumumab. These drugs target the extracellular domain of the EGFR and block down-stream signaling. Clinical trials for both agents initially required the pre-selection of patients based on positive expression of the EGFR protein, as determined by IHC, however, it soon became evident that IHC-based protein expression levels did not correlate with therapy outcome (32, 33) and that even patients with EGFR-negative tumors may benefit from EGFR-targeted therapy (34, 35). The causes for this discrepancy may be of a technical nature and connected to the variability/sensitivity of immunohistochemical techniques, or they may be a direct consequence of biological determinants. For instance, metastatic tumors may have lost the expression of EGFR, rendering them unresponsive to therapy (36). In addition, Chung and co-workers reasoned that antibodies used within IHC-based detection systems were unable to discriminate between high- and low-affinity EGFRs. Consequently, the relative distribution of such high- and low-affinity EGFRs within colorectal cancer tissue may be crucial in determining the response to therapy (34). Furthermore, it has been noted that therapeutic antibodies that target EGFR may induce antibody-dependent, cell-mediated cytotoxicity, resulting in an indirect beneficial effect owing to the recruitment of cytotoxic immune cells such as monocytes and natural killer cells to the tumor (34).

While the immunohistochemical detection of EGFR expression did not prove to be decisive in determining the clinical response, promising data has been generated in support of using the EGFR gene copy number as a predictive biomarker for EGFR-targeted therapy (37). Nevertheless, in order to achieve definitive proof and to facilitate the development of standardized testing modalities further investigation is required. In NSCLC, EGFR is targeted primarily using the small molecule inhibitors gefitinib and erlotinib. In contrast to colorectal cancer, mutations within the EGFR in NSCLC are common and mutational testing is recommended for all NSCLC cases (38). The application of mutation-specific antibodies for this purpose is discussed below. A further distinguishing feature regarding the testing of EGFR in colorectal cancer and NSCLC is that in the latter, IHC positivity or high EGFR gene copy numbers showed no conclusive correlation with treatment response (38).

### V-kit Hardy-Zuckerman 4 feline sarcoma viral oncogene homolog

Immunohistochemistry provides an excellent tool for the differential diagnosis of gastrointestinal stromal tumors (GIST). This is largely due to the fact that greater than 85% of GIST test positive for v-kit Hardy-Zuckerman 4 feline sarcoma viral oncogene homolog (KIT), in contrast to the negative result generated by most other mesenchymal tumors (39, 40). Imatinib mesylate (Gleevec, Novartis, Basel, Switzerland) is a specific tyrosine kinase inhibitor that exhibits high therapeutic activity in patients with chronic myeloid leukemia by targeting the fusion protein BCR-ABL (41). Additional targets of imatinib mesylate include the platelet-derived growth factor receptor (PDGFR) and KIT. For patients with unresectable or metastatic GIST, a positive immunohistochemical staining for KIT was initially required as an entry criteria into clinical trials investigating the efficacy of imatinib mesylate (42, 43). Significant clinical responses were recorded and this revolutionized the management of advanced GIST, a malignancy that had previously failed to respond to conventional chemotherapy. Activating mutations within the KIT gene and, to a lesser degree, PDGFR, are commonly found in patients with GIST and depending on their location within the coding region of the respective gene, they are highly correlated with the likelihood of a response to imatinib mesylate treatment (44, 45). That notwithstanding, since a small percentage of patients with GIST do not express detectable levels of KIT or do not harbor mutations within KIT/PDGFR (44–46) IHC or mutational analysis should not be used to deny treatment with imatinib mesylate since these patients may still be sensitive to this therapy.

### Anaplastic lymphoma kinase rearrangements

In addition to EGFR, the anaplastic lymphoma kinase (ALK) represents a second therapeutic target in NSCLC. Chromosomal rearrangements involving the associated gene have been detected in approximately 5% of cases, most frequently resulting in the fusion to echinoderm microtubule-associated protein-like 4 (EML4) and the constitutive expression of a chimeric tyrosine kinase protein (47, 48). Second-line treatment of NSCLC patients with confirmed ALK rearrangements, using the small molecule inhibitor crizotinib, has recently been shown to significantly prolong progression-free survival compared to standard chemotherapy (49). The current “gold standard” for ALK rearrangement testing is dual-color break-apart FISH. However, interpretation of test results may be challenging since EML4 and ALK are located on the same chromosome, resulting in limited separation of the 5′ and 3′ probes. To define a positive test result, only signals separated by more than two signal diameters and/or single 3′ signals (correlating to the ALK kinase domain) should be counted. In addition, at least 50 cells should be reviewed with a positive signal detectable in at least 15% (50). The immunohistochemical detection of ALK has been considered as an attractive addition to routine FISH testing. Since ALK is not expressed in lung tissue unless driven by promoter rearrangement, a good correlation between IHC and FISH results and low IHC background staining have been reported. In addition, a number of studies confirm that IHC-negative cases are almost exclusively negative in FISH analysis and therefore indicate that IHC could be applicable as a quick and cost-effective screening tool for ALK rearrangements (51–54). FISH reflex testing for all IHC positive cases has been proposed, somewhat similar to the evaluation strategy for HER2 (55). Interestingly, the percentage of ALK rearrangement positive cells during FISH evaluation did not significantly correlate with the response to crizotinib (56). Nonetheless, comprehensive data on the correlation between the intensity of ALK staining as determined by IHC and treatment response is still lacking.

## Mutation-Specific Antibodies

The selection of patients for a targeted cancer treatment frequently relies on the detection of specific gene mutations. The routinely applied techniques are generally based on the isolation of chromosomal DNA from fresh, frozen or FFPE material and analysis can involve various techniques such as mutation-specific real-time PCR, direct sequencing, mass spectrometry, mismatch ligation assays, high-resolution melting curve assays, or denaturating high-performance liquid chromatography, among others. A common drawback relates to the fact that information on tissue morphology is lost and also that “contamination” of tumor material with normal cells may hamper detection or obscure the results. In addition, increased demands on sample size and quality and extra requirements regarding technology and expertise, associated with higher cost and expenditure of time, frequently apply. The development of mutation-specific antibodies and their application in routine IHC may provide a convenient addition to DNA-based profiling techniques.

### BRAF V600E

**Figure 1:**
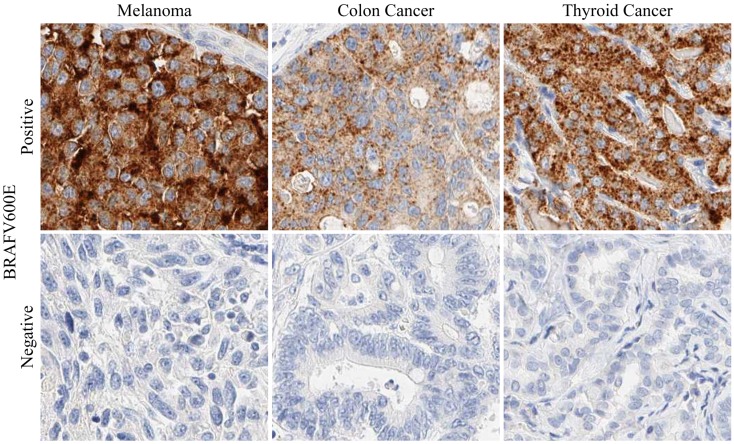
**BRAFV600E mutation-specific antibody staining**. Immunohistochemical staining examples of the BRAFV600E mutation-specific antibody VE1 are presented for a BRAFV600E-positive and a BRAFV600E-negative case of melanoma, colon cancer, and thyroid cancer, respectively. BRAFV600E-positive staining is generally detected as a granular, cytoplasmic signal that can easily be distinguished from BRAFV600E-negative cases. In the presented example images, 3,3′-Diaminobenzidine (DAB) was used as a chromogen.

The v-raf murine sarcoma viral oncogene homolog B1 (BRAF) represents an outstanding target for the development of a mutation-specific antibody. Mutations of the associated gene occur in a range of human malignancies including cutaneous melanoma, colorectal cancer, NSCLC, papillary thyroid cancer, and hairy-cell leukemia (57–59). By far, the most common BRAF mutation results in the substitution of valine for glutamic acid at position 600 (V600E), leading to the constitutive activation of the protein’s kinase domain. In human cutaneous melanoma, mutated BRAF has been detected in 40–50% of cases, with up 90% of these alterations concerning the V-E substitution at codon 600 (60). Vemurafenib and dabrafenib are two potent small molecule inhibitor drugs that specifically target BRAF V600E and have demonstrated remarkable response rates in metastatic melanoma patients (61, 62). Mutational testing of the patient tumor material is required before commencement of treatment and is to date commonly based on the detection of genomic alterations. As an addition to DNA testing, Capper et al. recently proposed a mutation-specific antibody for the detection of BRAF V600E in FFPE tissue specimens by means of IHC (Figure 1) (63). The results obtained from using this antibody to determine the BRAF mutational status in melanoma and thyroid cancer samples were identical to those achieved following DNA sequencing-based profiling. These results have since been substantiated by numerous studies with similarly high levels of specificity and sensitivity (up to 100%, respectively) (64–68).

In samples where the number of tumor cells is low, IHC-based BRAF testing was suggested to be more sensitive than direct DNA sequencing or high-resolution melting curve analysis (64, 65). In addition, IHC-based staining results showed low inter-observer variability (68). Nevertheless, despite the benefits of using IHC-based methods for determining the presence/absence of mutations within the BRAF gene, the presence of (non-specific) strong, nuclear staining may complicate the assessment of staining results. In addition, false-negative IHC results may occur due to unsuitable or incomplete tissue fixation, the presence of necrotic or pre-necrotic tissue areas or low levels of total protein expression (63, 64). The latter may be controlled for through the use of an additional antibody capable of detecting total BRAF protein expression. With regard to the intra-tumor heterogeneity of BRAF V600E, only few cases with non-homogenous expression have been observed following immunohistochemical detection (67, 69). These results are in direct contrast to previous reports describing significant variability of BRAF mutational status among individual cells within a tumor and warrant further investigation (70, 71). Regarding the possible relationship between BRAF V600E protein expression and clinical outcome, no significant correlation was seen between the percentage of BRAF V600E positive tumor cells and the response to treatment with either dabrafenib or vemurafenib (72). Similarly, the total intensity of mutation-specific antibody staining did not significantly correlate with patient outcome.

### EGFR L858R and E746_A750del

Epidermal growth factor receptor mutations have been detected in 2–17% of NSCLC patients from Europe and the United States, however, the mutational frequency increases to 30% when analyzing cases from East Asia (73–75). Specific mutations, in particular those affecting the EGFR kinase domain, have been associated with response to gefitinib and erlotinib treatment (76, 77). The two most common types of EGFR mutations are in-frame deletions of exon 19 and a leucine to arginine substitution at codon 858 (L858R) in exon 21. Taken together, alterations at these sites account for up to 90% of all EGFR mutations (78). Exon 19 deletions may affect a varying number of nucleotides. For example, E746_A750del results in a five amino acid deletion in the corresponding protein and is the most common deletion detected, occurring in approximately 70% of cases (79). The application of mutation-specific antibodies designed to target L858R and the E746_A750del modification, have yielded varying levels of detection specificity and sensitivity. For L858R, several studies reported sensitivity values in the range of 70–100% and specificities exceeding 95% (79–83). While these results were promising, sensitivities as low as 36 and 40% have also been described for the same antibody clone (84, 85). Similarly, regarding the E746_A750del-specific antibody, the same studies published sensitivity and specificity values of 40–100% and 95–100%, respectively. The possible sources of variation are numerous and include the application of different scoring systems, discrepancy between the definitions of positivity/negativity, different DNA-based reference techniques, different tissue fixation methods, and the types of specimens analyzed. While initial attempts to determine optimal tissue preparation and staining evaluation have been presented (86), additional steps toward a standardized protocol for the detection of EGFR mutations using IHC should be undertaken.

The overall high levels of specificity associated with IHC-based EGFR-mutational testing imply that IHC may be suitable as a pre-screening tool for the identification of NSCLC patients that are eligible for EGFR-inhibitor treatment. Since a number of mutations are not currently detectable by antibody-based profiling, the additional testing of IHC-negative cases using direct DNA sequencing or similar assays is necessary (79, 81). The applicability of IHC in predicting response to EGFR-targeted therapy remains controversial. Confirming the importance of EGFR-mutational status for treatment response, positive IHC staining has been associated with longer progression-free survival compared to IHC-negative or -equivocal cases (83, 87). In addition, high mutant EGFR expression (as defined by the sum of scores for fraction and intensity) was significantly related to elevated progression-free survival but not overall survival (88) and a fraction of positive tumor cells exceeding 50% of all cells predicted better response to EGFR inhibition treatment in univariate but not multivariate analysis (89). Despite the aforementioned results, a study by Kato and co-workers could not detect a significant correlation between IHC staining and treatment response or survival (82). Shortcomings in the significance of IHC-based detection methods in predicting survival benefit, in particular when compared to DNA-based techniques, may occur due to the limited mutation spectrum detected via IHC. Furthermore, EGFR-mutation-specific antibodies have been shown to occasionally detect mutations associated with EGFR-inhibitor resistance via mechanisms that are not yet fully understood (84).

## Alternative Protein Binders

**Figure 2:**
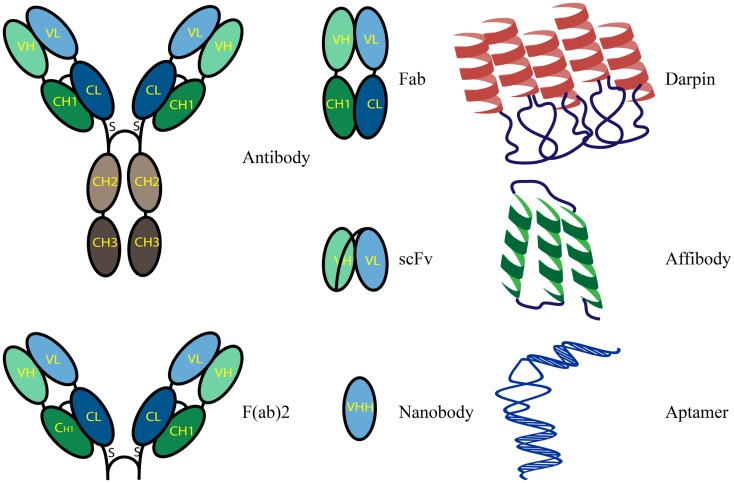
**Schematic representation of binder formats**.

*In situ* affinity-based detection of proteins remains one of the best sources of information about either the healthy status of an individual tissue or potential pathological changes, and is thus applicable within several medical settings. Molecular imaging allows for the early detection and classification of many human diseases and, when specific, permits improved, target-directed therapies. Molecules generated through immunization such as polyclonal, monospecific polyclonal, and monoclonal antibodies continue to be the best established and most widely used binders in diagnostics (90, 91). Methods for the detection of proteins based on antibody recognition often encounter problems due to poor selectivity and/or sensitivity (92). Poorly characterized antibodies and/or insufficient quality control often render them as unsuitable for demanding applications such as companion diagnostics (93). Commercially available antibodies frequently perform very differently within various laboratories and often do not perform as advertised, thus raising doubts regarding their reliability when incorporated into assays requiring high specificity (94). Antibodies can be biochemically and physiologically modified and use of their derivatives, such as single chain variable fragments (scFv) or Fab fragments, may result in the improved detection of a wide range of target molecules (Figure 2) (95). Currently only a few antibodies and recombinant proteins are used within clinical settings, largely due to the reasons outlined above. Recombinant binders that are generated in immune-free, *in vitro*-based approaches, hold the potential of taking priority over conventional antibodies (96). Stability, specificity, ease of manipulation, low cost, high throughput, and reproducibility of production are some of the advantages that make novel scaffold molecules highly desirable (97). Alternative binders are promising molecules for novel approaches in individualized medicine (Figure 2). They can serve as personalized molecular imaging tools for *in vivo*, live diagnostics of the changes occurring in the expression of markers following treatment (95).

### Recombinant antibody fragments

Antibody fragments are generated in order to obtain binders with improved characteristics, compared to conventional antibodies, but similar functional and recognition properties. Fab and F(ab)_2_ fragments are antigen-binding fragments generated after proteolysis of full length antibodies. Single chain variable fragments (scFvs) are smaller than Fab fragments and are composed of genetically linked light and heavy chains containing variable regions. These molecules can also exist as dimers (98). Another class of antibody-derived antigen-binding molecules, termed nanobodies, are small, single-domain polypeptides derived from the variable part of the heavy chain (VHH) of light-chain deficient antibodies that were originally discovered in camelids (camels, llamas) (99, 100). Their reduced size, greater stability and solubility, and antibody-like binding characteristics make nanobodies ideal for use in the targeting and imaging of antigens in live cells, protein precipitation *in vivo*, and targeted enzymes modulations (101–103). As a result of their smaller size (only 15 kDa), nanobodies can bind to epitopes that are hidden or shielded and reach affinities within the range of nanomolar to picomolar. Nanobodies are highly specific for their targets and have no known cross-reactivity to structurally related proteins, which makes them excellent tools for targeting kinases and tyrosine phosphatases. In addition, nanobodies have technological advantages that render them superior to conventional antibodies. They are easily modified to avoid chemically reactive groups such a primary amines or to alter the primary amine number to allow more selective and controlled chemical conjugation. The selection of binders for certain applications is usually based on their performance in experimental settings and depends on their preference for particular epitopes and the accessibility of binding sites.

### Affibody molecules

Affibody molecules are small ( ∼7 kDa), alpha-helical *Z*-domain of Staphylococcal protein A, immune-independent affinity molecules that target a wide range of proteins (104, 105). They can be produced in functional form both via recombinant expression in *Escherichia coli* or peptide synthesis. They possess picomolar affinities, are highly soluble and stable. In addition, they are cysteine-free which prevents non-specific binding events when applied to tissues. Furthermore, the lack of cysteine provides an opportunity for site-specific labeling through the introduction of unique cysteine molecules. High affinity affibodies were engineered against targets such as the IL2 receptor, Alzheimer’s amyloid-beta peptide or EGFR (106–108). They have been used in various types of experiments and are intended for both *in vivo* and *in vitro* imaging and also therapeutic applications including the detection of HER2 within different experimental settings (109, 110).

### Designed ankyrin repeat proteins

Designed ankyrin repeat proteins (DARPins) are potent alternatives to conventional antibodies. They detect antigens with high specificity and picomolar affinity, are independent of target immunogenicity and possess attractive molecular properties such as small size and high stability (111). They are synthetic, non-immunoglobulin binding proteins that form scaffolds containing tandem repeats of an elementary, structural motif, typically composed of 33 amino acid residues folded into a β-turn followed by two antiparallel α-helices. A single protein may contain up to 29 repeats of this motif. The production of DARPins does not require the use of animals at any step, therefore permitting the large scale, parallel production of variable binders. DARPins are correctly folded in both prokaryotes and eukaryotes, due to the absence of disulfide bonds, which enables their use in a variety of functional assays (112). They can easily be genetically modified to form fusion proteins and site-specifically targeted for chemical conjugation. DARPins against a wide range of protein targets, including extracellular, intracellular, and membrane proteins, were generated with high yield from synthetic libraries and successfully used as replacements for conventional antibodies. One such example is the DARPins generated against HER2; these displayed higher specificity and similar sensitivity when compared to FDA-approved antibodies for the *in situ* identification of HER2 expression status in FFPE breast cancer tissue (113). DARPins can easily be made functional for use in various biomedical applications through the introduction of site-specific, clickable modifications. Such alterations do not affect their physical properties (114).

### Aptamers

Aptamers are a class of small, synthetic, self-folding, and single-stranded RNA or DNA molecules that form secondary and tertiary structures and specifically bind to proteins, small molecules, or other cellular targets such as nucleic acids (115, 116). They are comparable to antibodies in terms of their target recognition capabilities, their binding affinities, and the diversity of applications that they can be used in; however, they possess numerous significant characteristics that render them advantageous over their protein equivalents. Aptamers are highly specific, non-immunogenic, redox-insensitive, and temperature- and pH-tolerant. In addition, they do not have hydrophobic cores which are usual in proteins and, therefore, they do not aggregate. In order to select aptamers, information on protein conformation is not required, a feature which can be useful for screening for unidentified disease biomarkers. Aptamers can be generated through cell-based aptamer selection that utilizes differences between the molecular signatures of any two different cell types. The selected aptamers selectively bind to an unknown protein within one cell type only, are cross-linked to their targets and once the complex is purified the targets can be analyzed by mass spectrometry (117). Therefore, the cell-based selection of aptamer molecules has great potential for the development of specific probes suitable for biomarker discovery and companion diagnostics development. Since chemical synthesis is a process that is well defined and highly reproducible, the production of aptamers can easily be scaled up. They can be synthesized with specific, custom tailored functional groups attached to 5′ or 3′ termini which creates an easy approach to conjugation and multiplexing *in situ* assays. Furthermore, aptamer conjugations do not generally alter their binding affinity. With advances in imaging techniques, aptamers are already considered as prospective reagents for *in situ* targeting. To date, several aptamers have been developed against important clinical targets such as PDGF, von Willebrand factor (vWF), E-selectin, vascular endothelial growth factor (VEGF), and prostate specific membrane antigen (PSMA) and their applicability within a clinical setting is currently being investigated (118–121).

## *In situ* Proximity Ligation Assay

**Figure 3:**
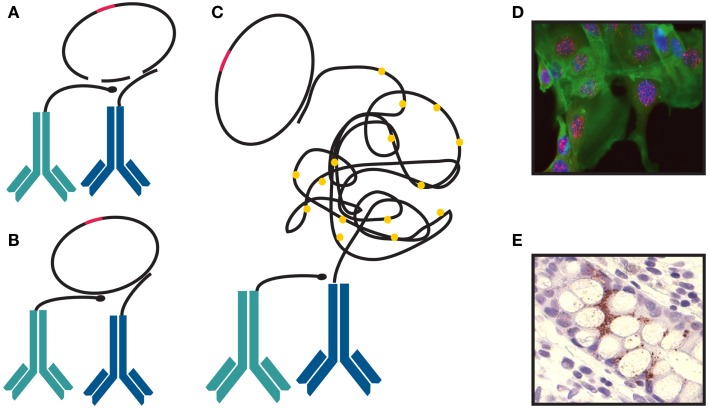
**Proximity ligation assay**. **(A)** Two probes stay in close proximity by binding to a protein or two proteins present in one complex. **(B)** They are joined and circularized by DNA ligation upon introduction of linear connector oligonucleotides. After ligation, rolling-circle amplification (RCA) is initiated. One of the proximity probes is used as a primer. **(C)** The single-stranded RCA products are hybridized with labeled detection oligonucleotide complementary to a multiplied motif in the sequence of the RCA product. The detection oligonucleotide can be labeled with fluorophore **(D)** or a horse radish peroxidase **(E)**.

Using traditional IHC techniques, the level of protein expression can easily be determined. However, the functional status of a cell cannot be evaluated by the level of expressed protein alone. The activity of signaling pathways, as assessed by the analysis of post-translation modifications (PTMs) and protein interactions, needs to be determined and taken into consideration (122). Cancer does not consist of a homologous mass of cells but of complex, heterogeneous cell populations that are affected by interactions with each other and the surrounding environment. Therefore, the analysis of cancer tissue at single cell resolution provides a much better understanding of the differences in signaling status and activity (123). *In situ* PLA enables a localized and specific detection by utilizing oligonucleotide-conjugated antibodies to determine the proximity between one or more targeted epitopes. This makes it a suitable method for detecting molecular events in cells and tissue, for example the status of a signaling pathway. The use of two independent binders and the additional requirement of proximity for reporting enable the specific detection of proteins, protein–protein interactions, and PTMs (124). Proximity ligation converts the recognition of a protein, protein complex, or PTM by two or more antibodies into an amplifiable, circular DNA molecule (Figure 3). Upon proximal binding of a pair of oligonucleotide-conjugated antibodies (PLA probes), the oligonucleotides guide the formation of a circle after applying two additional, single-stranded DNA molecules. This circular DNA molecule is then ligated and amplified by phi29 polymerase within a rolling-circle amplification (RCA) reaction, resulting in a localized, concatameric product. The latter is visualized by hybridization of detection oligonucleotides labeled with fluorophores or horse radish peroxidase (Figure 3) (125). Due to the environment of fixed cells and tissue the amplification product will collapse into a bundle with a diameter of approximately 1 μM (126) that can then be visualized as a bright dot that is quantifiable and easily distinguished from the background (127).

Heterogeneity within a sample increases the demands on the dynamic range of a method in order to allow for the detection of both abundant and scarce targets within the same sample. *In situ* PLA uses an amplifiable DNA circle as a reporter molecule and by using reagents that give rise to three variants of the reporter DNA circles and adding them at decreasing concentrations the dynamic range of the PLA is increased. By labeling the circles with different fluorophores, the readout can be adjusted to the fluorophore whose concentration gives rise to quantifiable and easily distinguishable signals. In a heterogeneous sample, different readout fluorophores can be used for different parts of the sample, enabling the detection of a target that varies greatly within the sample without the risk of signal saturation. As the size of a patient sample is often limited, this approach reduces the need to optimize the binder concentration and enables the evaluation of patient samples where knowledge on the expected results is limited (128).

Multiplex *in situ* PLA permits the parallel analyses of multiple protein complexes involved in signaling pathways directly in tissue and cells thus making it possible to compare levels of protein complexes between individual cells and also providing information regarding the spatial distributions of these complexes. A tag-specific sequence within the PLA probe targeting a protein gives rise to a DNA circular molecule that carries information on the identity of the target protein. The amplified tags in the RCA products can then be visualized using oligonucleotides labeled with different fluorophores, to uniquely recognize the tag sequences corresponding to a certain target (129). *In situ* PLA has been shown to provide valuable information about the status of signaling pathways by detecting molecular events such as dimerizations, the formation of protein complexes and PTMs. It enables the detection of activity at different levels within a signaling pathway, thereby enabling specific aberrations to be pinpointed. *In situ* PLA has been utilized in studies investigating both EGFR dimerization and receptor activation, which has been proposed to play a crucial role during tumor progression, and also the development of drug resistance. Dimerization and aberrant activity has been shown to be independent of EGFR expression, explaining why the deregulated expression of the EGFR in several types of human malignancies was shown to have limited value as a prognostic or diagnostic marker. Receptor dimerization events detected by mutation-specific PLA appeared to be more suitable for the selection of patients for EGFR-targeted treatment (130).

Similarly, the overexpression of human epidermal growth factor receptors (HERs) has been linked to poor prognosis in patients with early breast cancer. Dimers containing the HER2 isoform were shown to be more stable and have prolonged active signaling compared to HER2 deficient dimers. Elevated levels of HER2-HER2 and HER2-HER3 complexes detected by PLA showed a significant association with decreased recurrent-free survival and a reduction in overall survival of breast cancer patients, proving that PLA and the detection of cellular signaling processes can be successfully implemented in studies on prognostic markers in clinical specimens (131). Through the application of *in situ* PLA, it is now possible to screen for the effects of a drug treatment on intracellular signaling, providing information on the specific level of signaling pathways. Being able to study primary cell lines and patient tissue sample gives valuable information of the signaling status within a specific tumor and allows to predict the response to a certain therapy (132). PLA technologies have been used to address a variety of biomedical problems and demonstrated the potential to address some difficulties, both concerning the validation of biomarkers and the applicability for clinical diagnostics. Implementing PLA techniques as an alternative to IHC in everyday laboratory practice allows for a more precise and quantitative evaluation of antibody performance characteristics and their suitability for an anticipated analytical use. Application of the PLA technique provides an opportunity to develop a high-quality procedure for *in situ* detection of proteins and signaling pathways in companion diagnostics. This will offer the medical industry powerful, universally applicable tools for clinical research and routine diagnostics.

## Conclusion

Affinity proteomics for the analysis of proteins as companion diagnostics requires access to reagents that can be used in specific detection reactions. The comprehensive validation and improvement of existing and newly generated antibodies to obtain well characterized, high-quality, and well-controlled resources as tools for large scale studies of the human proteome in health and disease is a widely acknowledged demand. In many routine clinical, diagnostic, and life science applications, antibodies have proven to be the reagents of choice. IHC is routinely performed in the majority of clinical laboratories and widely acknowledged as superior to other analysis techniques regarding time- and cost-effective application. In selected cases, IHC and mutation-specific antibodies may even provide an attractive alternative to DNA-based testing methods. As a highly valuable resource documenting the availability and identification of novel biomarker candidates, the Human Protein Atlas^2^ project has generated antibodies targeting proteins from over 15,000 genes, corresponding to about 75% of all human protein-coding genes (133, 134). All antibodies are routinely subjected to a series of validation steps, including protein arrays, western blots, and immunofluorescence and used to assess protein expression patterns in a broad spectrum of normal and cancer tissues through application of IHC. Nevertheless, efforts to detect proteins when high specificity is required often fail. Hence, there is a strong need for better methods and reagents for assessing protein expression in tissues as means of companion diagnostics and alternative binders or PLA in combination with existing antibodies represent promising candidate alternatives.

## Conflict of Interest Statement

The authors declare that the research was conducted in the absence of any commercial or financial relationships that could be construed as a potential conflict of interest.

## Footnotes

^1^http://www.fda.gov/MedicalDevices/ProductsandMedicalProcedures/InVitroDiagnostics/ucm301431.htm

^2^www.proteinatlas.org
